# Filling the Gaps in Oncofertility Care by Addressing Challenges Faced by Patients and Providers

**DOI:** 10.7759/cureus.50219

**Published:** 2023-12-09

**Authors:** Saanthwana Ranjith, Apoorva Dave

**Affiliations:** 1 Obstetrics and Gynecology, Jawaharlal Nehru Medical College, Datta Meghe Institute of Higher Education and Research, Wardha, IND

**Keywords:** doctor-patient communication, refferal networks, ethical and social perspectives, fertility preservation, oncofertility

## Abstract

With enhanced technology and upcoming treatment strategies in the cancer field, the survival rates of patients have increased. We have now reached a stage in the treatment of cancer where we not only address the disease but also address complications that arise due to the disease and the side effects that present in the post-survival population due to its treatment. One of the primary consequences after oncotherapy is infertility, which is a major reason for distress for patients' post-survival, as they are afraid they may be deemed as less desirable, be rejected by their existing partner, or cannot grapple with the fact that they cannot have children of their own. This can be avoided by the implementation of proper oncofertility practices. The subject of oncofertility involves interactions between experts in the domains of cancer diagnosis, therapy, fertility preservation, and reproductive health. It attempts to investigate and broaden the possibilities for cancer survivors' reproductive future in order to suit their needs according to their ethical religious and sociocultural beliefs. However, these practices are often not implemented effectively due to ineffective doctor-patient communication, lack of knowledge, or partial knowledge of clinicians themselves regarding fertility care. This leads to a feeling of insecurity among clinicians hence resulting in them not referring patients. Lack of awareness among doctors of different oncofertility procedures available especially for patient groups like women and younger patients, hence leading to reduced referral in these groups. Improper coordination across health departments, patient ignorance regarding procedures, financial instability especially in a country with a lower sociodemographic index like India, and neglect or less importance given to the related ethical, social, and legal issues. In this article, we cover the effects of cancer and cancer treatment on fertility, the options available to adult and pediatric cancer patients to preserve their fertility like oocyte/ovarian tissue cryopreservation in females and sperm cryopreservation in males, techniques undergoing experimental studies that could be implemented in the future like spermatogonial stem culture and transplantation of testicular tissue, the obstacles that we face that hinder the proper implementation of such practices and what measures can we take to overcome these obstacles to improve patient care and be better healthcare providers.

## Introduction and background

Around the globe, cancer incidence is about an estimated 19.3 million [1]. With the recent advancements in medicine, we’ve progressed from a stage in cancer treatment and diagnosis, where survival was the only goal, to a stage where we consider how to improve the quality-of-life post-cancer survivorship. A common consequence of cancer and cancer therapy is infertility. In an ideal situation, the effect of the disease or its therapy on future reproductive and endocrine health should be included in the initial plan of care provided to patients, but this decision to protect fertility is made difficult by various factors like how old they are, if they’re married or not, if they can postpone treatment and their chances of survival [2]. Oncofertility is a discipline that is an amalgamation of interactions between fields like diagnostics, cancer therapy, fertility preservation, and reproductive health. It aims to explore and broaden the horizon of options available for the future reproductive health of cancer survivors in order to meet their needs [3]. However, we should not forget that not only does this field require advancements in fertility preservation techniques, but it should also address the ethical, social, and legal aspects associated with it [4]. Proper execution of fertility care before the initiation of cancer treatment requires effective communication between consultants with the patient and their families before undergoing treatment, in order to facilitate this process healthcare workers should themselves be equipped with knowledge regarding fertility preservation techniques to ensure timely referral. In this article, we discuss the impact of anti-cancer drugs and radiotherapy on fertility, along with the choices available for adult and pediatric cancer populations to preserve their fertility, the legal hurdles faced in providing care, and the role that clinicians from various branches may play in removing those hurdles. By analyzing the common cancer types and the ones requiring aggressive therapy in Table 1, we can focus on what types of cancer should special attention be paid to in terms of fertility preservation [5-13].

**Table 1:** Epidemiology of common types of cancer in different age groups that require gonadotoxic treatment

Age group	Female	Male
Prepubertal (0-14 years)	Leukemias, Central nervous system, Lymphomas, Renal	Leukemias, Central nervous system, Lymphomas, Renal
Adolescents and young adults (15-24 years)	Lymphoma, Melanoma, Central nervous system, Leukemias	Germ cell tumors, Lymphoma, Central nervous system, Leukemias
Adults (25-49 years)	Breast, Melanoma, Cervical, Central nervous system, Ovarian	Testis, Melanoma, Lung cancer, Central nervous system, Head and neck
Most common types of cancer that may require aggressive chemotherapy and necessitate prior fertility preservation measures	Breast, Cervical, Leukemia, Lymphoma Central nervous system	Testicular, Germ cell tumor, Leukemia, Lymphoma, Central nervous system

Understanding how cancer treatment affects fertility

In women, the ovarian follicular reserve can be rapidly depleted due to the action of chemotherapeutic medication on the ovary's biological components. Cyclophosphamide, cisplatin, and doxorubicin are medications often used in cancer treatment, these medications cause early ovarian insufficiency by causing the death and/or rapid activation of primordial follicles and increased degeneration of developing follicles. Additionally, they exacerbate inflammation and injury to blood vessels and the stromal compartment [14]. In young women who have had radiation therapy, the danger of ovarian failure and considerably hampered uterine development and blood flow are among the reproductive issues faced, the magnitude of these issues is influenced by the field of radiation, overall dosage, and fractionation schedule [15]. In men, alkylating chemotherapeutic drugs like cyclophosphamide and busulfan target spermatozoa since they are rapidly replicating cells and often cause azoospermia [3]. The damage imposed on spermatogenesis by radiotherapy is proportional to the same variables as observed in females.

## Review

Material and methodology 

**Figure 1:**
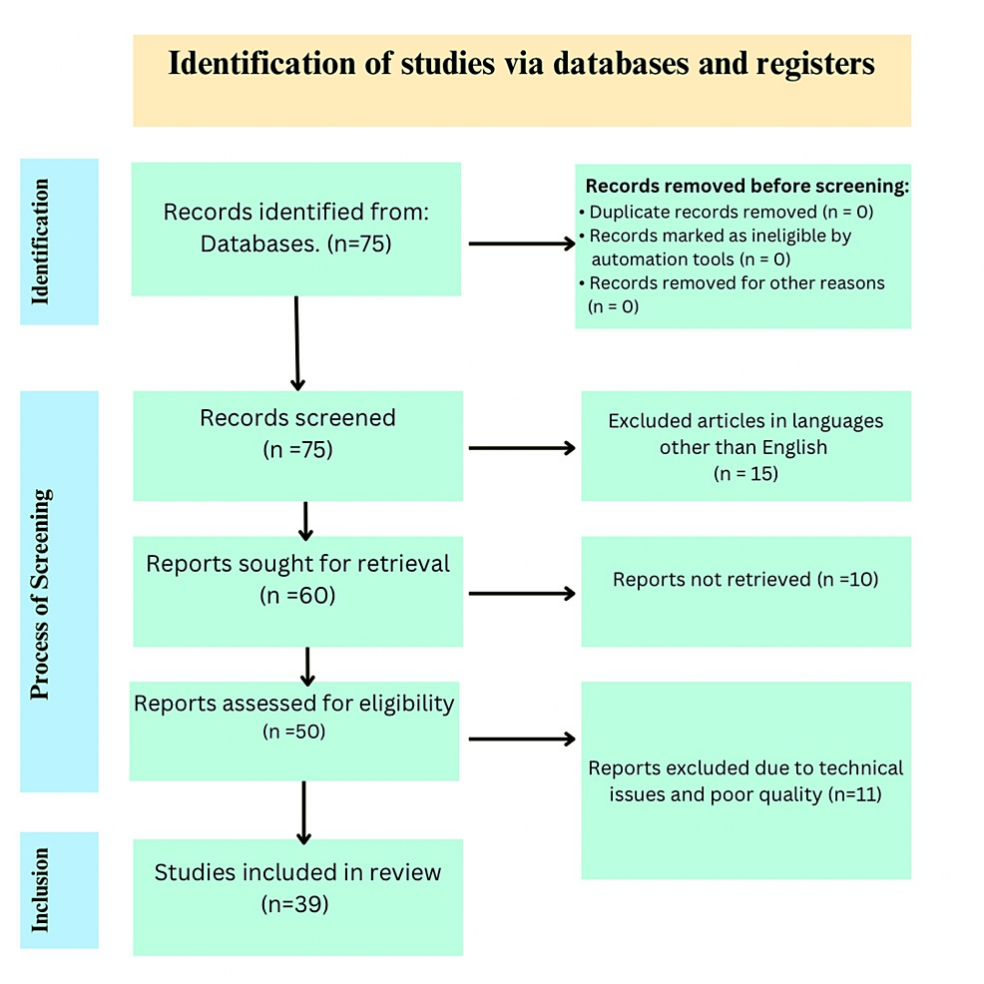
PRISMA flow diagram for the selection of review materials PRISMA: Preferred Reporting Items for Systematic Reviews and Meta-Analyses

A detailed search was done on PubMed till August 2023. Advanced Medical Subject Headings (MeSH) terms, such as fertility preservation, oncofertility, ethical and social perspectives regarding oncofertility, doctor-patient communication, and referral networks were used interchangeably and in combination. The inclusion criteria consisted of articles that discussed oncofertility procedures, ethical and social aspects related to it, and awareness among doctors and patients regarding the procedures, articles in the English language, and for which PubMed or the publisher provided open access. The articles that were excluded were articles that were in languages other than English and were not retrievable (i.e., not having open access). A total of 75 articles were found, but only 39 of them were chosen to be included because it was determined that they were pertinent. These were selected following the Preferred Reporting Items for Systematic Reviews and Meta-Analyses (PRISMA) guidelines. A comprehensive outline of the selection method is given in Figure 1.

Options for fertility preservation

Fertility preservation is attributed to any surgical and medicinal procedures intended to lessen the action of cancer therapy on future fertility [16]. Standard methods for women include oocyte and embryo cryopreservation. This method requires 12-14 days for hormone stimulation for the retrieval of oocytes. A disadvantage to this method is that it can be conducted only in women who have achieved puberty. Therefore, it cannot be used in children who do not have mature oocytes available. Another concern is that the hormone stimulation period required for this procedure comprises two to five weeks; two problems arise from this: not all patients can postpone treatment for that long, and exposure to hormones during the period of stimulation might cause hormone-positive cancers to progress faster [17]. For the pediatric age group, techniques like ovarian tissue cryopreservation are available, which involves surgically collecting the tissue (ovarian cortex), which is then frozen. Once cancer therapy is completed, the tissue is thawed and placed back into the ovary [18]. A faster option for those dealing with time and hormone-sensitive cancers is in vitro maturation (IVM), which involves completing nuclear and cytoplasmic maturation of retrieved immature oocytes outside the body and in vitro follicle growth (IVFG), which involves maturation of follicles outside the body. The methods can apply to pre-pubertal females as well [19]. Ovarian transposition is a technique where we detach the ovaries and fallopian tubes from the uterus and attach them away from where the radiation is focused, i.e., the abdominal wall. However, it is not always an efficacious method, as the ovary might travel back and get exposed to radiation [20].

**Figure 2:**
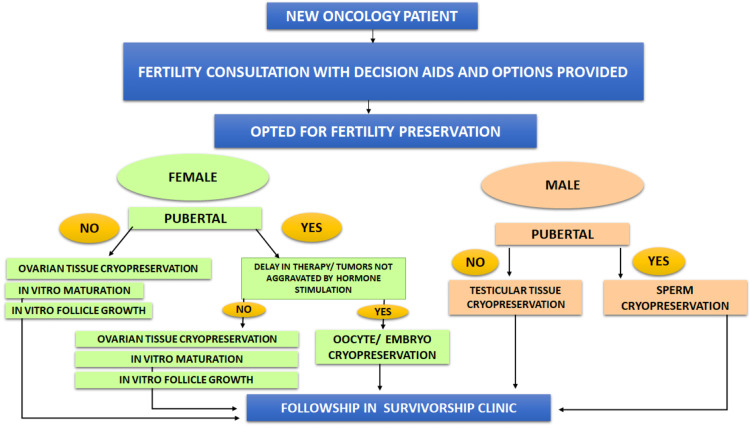
Overview of available fertility options Image credits : Saanthwana Ranjith

Standard methods for adult and post-pubertal males include sperm cryopreservation. First, semen analysis must be conducted in these men; if sperm is found in the ejaculate, then cryopreservation should be followed (about 10-12 vials are collected) [21]. Five phosphodiesterase inhibitors, along with intercavernosal injections, are prescribed in patients who are unable to ejaculate due to an erectile dysfunction [22]. In patients who are unable to produce ejaculate or have complaints of retrograde ejaculation, methods like penile vibratory stimulation (PVS) and electroejaculation (EEJ) can be used to collect semen for cryopreservation [23,24]. Techniques in prepubertal males are still undergoing studies, with experimental studies on animal models trying to explore cryopreservation of testicular tissue and spermatogonial stem cell culture and transplantation [25,26]. A rough overview of the fertility options, as mentioned earlier, is given in Figure 2.

Ethical barriers faced in oncofertility care

Many physicians often find it difficult to advise patients regarding fertility preservation procedures while informing them about their cancer diagnosis; they are often torn about the fact whether it is ethical to talk about their reproductive status when the patient’s own life is at stake. An important thing to note is that sometimes news regarding infertility might be just as upsetting for patients as their cancer diagnosis, as noted by their association with conditions such as anxiety and depression. By hearing about their impending loss of fertility, the patient may also fear that their partner might leave them, or they won’t be seen as desirable as they are unable to bear a child, which is superimposed by preexisting psychological distress due to cancer diagnosis and treatment [27]. Another concern among bioethicists is that should cancer patients opt for parenthood as their unlikely demise or compromised health status could leave the child unattended. However, a good point to consider is that patients who have cancer and opt to preserve their potential to give birth are just protecting their rights. An interest in fertility preservation techniques also shows that they are optimistic in the face of adversity despite a probable unfavorable diagnosis. It could be a coping mechanism that could keep them going while combating the condition since it gives them hope that they might have a normal life after treatment. These choices are more complex in the case of children and adolescent age groups. A few concerns regarding this age group are: the child might not be able to make an informed decision due to their inability to fully understand the situation and its consequences. Secondly, as children undergo maturation at different rates, there are no guidelines based on age that specify what definitive age group of children can make an autonomous decision regarding their reproductive health. This might implicate the role of parents in choosing options, which may complicate the process. Thirdly, physicians might hesitate to provide counseling regarding fertility preservation, as the parents might find it uncomfortable to view the possibility of the child as a sexually mature human being [28]. Finally, in the future, in the case of good survival outcomes and where the parents had opted for preservation techniques, the child might have this burden of giving birth to offspring because their parents have invested so much financially and economically to preserve their ability to give birth [29]. Another concern regarding parental consent is: Should refusing to opt for fertility preservation be considered an equivalent to sterilizing their child? We should realize parents have the right to consider their values while making an informed decision for their child. Also, often, they might prioritize their child’s health over their reproductive potential [30]. The financial aspect is another thing that should be considered. Cancer treatment is already an emotional and economically draining process, so suggesting fertility preservation techniques to patients who can barely afford cancer treatment could be considered quite insensitive. Many patients might not opt for preservation techniques for sociocultural or religious reasons.

Challenges faced by physicians and patients

The reasons why most physicians or oncologists fail to provide timely referrals to their patients is due to a lack of knowledge about fertility preservation options and the fact they are not updated about the recent advances in the field. It is also because they are not aware of where exactly to refer patients. These two barriers play a vital role in missed referrals since, without the physician notifying them, the patient would not be aware of the options available to them. Other barriers to the discussion regarding preservation practices include where oncologists would prioritize treatment and would not recommend delaying treatment. Oncologists also find it difficult to ensure proper communication regarding such practices owing to the high patient load and limited time available [31]. Another issue that arises is due to miscommunication between specialists; for example, it is assumed that the brunt of responsibility is of the oncologist; however, in some cancers like breast cancer, the patient would be referred to a surgeon for their treatment and later referred to an oncologist. Now, due to some misunderstanding, the surgeon might assume that fertility preservation will be talked about by the oncologist and vice versa; therefore, the discussion does not take place at all [32]. Even if patients sign forms before chemotherapy stating that they are aware of the consequences faced after therapy, one being infertility, it does not warrant proper counseling regarding the matter [20]. Therefore, highlighting the need for proper framework guidelines and counselling. Patient concerns include information healthcare professionals provide being incomplete or too much to process in one sitting [33,34]. Financial problems, anxiety regarding postponing treatment, not favoring fertility preservation options due to a lack of awareness regarding the matter, or misinformation spread regarding the matter. Patients may not pursue the same for various ethical and social reasons. According to a study conducted in North India, the following referral practices were followed by healthcare workers (Table 2).

**Table 2:** Referral practices were followed by healthcare workers according to a study in North India [35]

Referral practices	Percentage (n = 150)
Routinely referred patients for counselling	53%
Didn’t refer due to lack of knowledge	43% (n = 58)
Didn’t refer due to financial burden on the patient	22.5% (n = 30)
Didn’t refer due to ignorance about referral pathways	16.5% (n = 22)
Didn’t refer due to other reasons like transmission of cancer to offspring, unmarried status and lack of time	Least common

Measures to combat hurdles faced in care

To combat the lack of awareness regarding oncofertility practices among clinicians, we can start oncofertility clinics, which comprise oncologists and reproductive medicine specialists. This helps us to formulate procedures for patients, and it also helps to evaluate cancer type, risk of toxicity of drugs to the reproductive organs, the desire of the patient to opt for preservation techniques, and if the patient is fit enough physically and mentally to accept treatment. Since doctors who are more knowledgeable about fertilization preservation practices are likely to refer patients, we can increase awareness among doctors by conducting seminars regarding new technology, try including it in the curriculum for doctors specializing in these fields, and circulate leaflets and newsletters so that cancer clinicians can be oriented to such practices on a national level [36]. Improper counseling due to a lack of time can be addressed by promptly referring patients to reproductive medicine specialists for counseling when they receive their initial diagnosis. Nursing staff and other patient navigators can also be trained to discuss any inquiries regarding fertility procedures that patients have, and they can aid the process by providing counseling in the initial phases and increasing referral rates [37]. Financial concerns can be addressed by the government to provide gamete/embryo cryopreservation at minimum cost to patients who are unable to bear the financial burden of such procedures. Awareness can be built at the national level by the utilization of social media, which can provide a platform for patients undergoing procedures to network and be aware of advancements in technology available at their disposal. Patients can be further provided pamphlets and written content (in their local language) during treatment and counseling to improve their knowledge and make informed decisions [38]. In addition, medical societies and bodies at the national level can organize workshops and conferences that can spread information regarding such practices. In relation to other concerns like religious and ethical fronts, although clinicians cannot be expected to be specialized or have similar spiritual views as that of their patient population the least they can do is understand religious beliefs can play an integral role in opting for oncofertility care, ensure that patient receives appropriate counseling resources which helps them choose an option which goes along with their beliefs and help them formulate a treatment plan which respects these principles of the patient [39]. The gap between cancer and fertility can, however, only be bridged by developing a multidisciplinary network of clinicians across various specialties namely oncology, hematology, reproductive endocrinology, urology, surgery, pathology, and healthcare workers which includes nursing staff and psychologists [35]. Table 3 summarizes obstacles faced and proposed solutions.

**Table 3:** Reasons for improper referral and solutions to correct it.

Reasons for improper referral	Solutions
Miscommunication across specialities	Integrated oncofertility clinics, multidisciplinary networks like oncofertility consortium
Doctor insecurity or lack of knowledge regarding procedures	Enhance knowledge through workshops, adding it to the curriculum
Lack of time / too much information in one sitting	Patient navigators and decision aids
Financial concerns	Government/non-governmental organizations can initiate programs at lower cost
Patient ignorance	Connecting to other patients with similar ailments via social media, decision aids
Religious and sociocultural reasons	Appropriate counselling which helps the patient choose the option that goes along with their beliefs. Local language aids and an interpreter can also aid in the process.

The endpoints of the articles that were included in our review are given in Table 4.

**Table 4:** Details of individual studies that are included

Sr no.	Author	Year	Endpoint	Type of study	Publishing Journal
1.	Sung H, et al. [1]	2021	Statistics on incidence of cancer.	Review article	A cancer journal for clinicians
2.	Woodruff T, et al. [2]	2007	Fertility preservation in cancer patients and factors that affect it.	Review article	Springer journal
3.	Halpern JA, et al. [3]	2020	Definition of oncofertility and gonadotoxicity of chemotherapeutic medication in males.	Review article	Translational urology and andrology
4.	Woodruff TK, et al. [4]	2010	Discussing fertility outcomes in young cancer patients.	Review article	Nature reviews clinical oncology
5.	Devine SM, et al. [5]	1994	Mentioned in table which summarizes epidemiology of common types of cancer which require gonadotoxic treatment	Review article	A cancer journal for clinicians
6.	Shoemaker ML, et al. [6]	2018	Mentioned in table which summarizes epidemiology of common types of cancer which require gonadotoxic treatment	Review article	Springer journal
7.	Miller KD, et al. [7]	2021	Mentioned in table which summarizes epidemiology of common types of cancer which require gonadotoxic treatment	Review article	A cancer journal for clinicians
8.	Kanas G, et al. [8]	2021	Mentioned in table which summarizes epidemiology of common types of cancer which require gonadotoxic treatment	Original article	Taylor and Francis online
9.	Libes J, et al. [9]	2023	Mentioned in table which summarizes epidemiology of common types of cancer which require gonadotoxic treatment	Special report	Wiley online library
10.	Arnold M, et al. [10]	2022	Mentioned in table which summarizes epidemiology of common types of cancer which require gonadotoxic treatment	Original Investigation	JAMA dermatology
11.	Bridges B, et al. [11]	2007	Mentioned in table which summarizes epidemiology of common types of cancer which require gonadotoxic treatment	Review article	Lippincott Williams and Wilkins, Inc
12.	Goana-Luciano P, et al. [12]	2020	Mentioned in table which summarizes epidemiology of common types of cancer which require gonadotoxic treatment.	Review article	Chinese Clinical Oncology
13.	Buskwofie A, et al. [13]	2020	Mentioned in table which summarizes epidemiology of common types of cancer which require gonadotoxic treatment.	Review article	Journal of National Medical Association
14.	Spears N, et al. [14]	2019	Gonadotoxicity of chemotherapeutic drugs on female reproductive system.	Review article	Human Reproduction Update
15.	Ho C, et al. [15]	2002	Effect of radiation therapy on female fertility.	Original Article	Taylor and francis
16.	Kondapalli LA, et al. [16]	2007	fertility preservation in oncofertility patients.	Chapter of a book	Springer Link
17.	Gracia C, et al. [17]	2012	oncofertility preservation techniques in females	Chapter of a book	Springer Science and business
18.	Fabbri R, et al. [18]	2012	Oncofertility techniques in female paediatric age group	Original research	Obstetrics and Gynecology International
19.	Bertoldo MJ, et al. [19]	2020	Fertility preservation procedures for adolescent age group	Review article	Elsevier
20.	Oktay K, et al. [20]	2018	Oncofertility techniques and ethical concerns.	Review article	An American Society of Clinical Oncology Journal
21.	Esteves SC, et al. [21]	2015	Oncofertility preservation techniques in males.	Review article	International Urology and Nephrology
22.	Burnett LA, et al. [22]	2018	Oncofertility preservation techniques in males (intracavernosal injections).	Review article	The Journal of Urology
23.	Castle SM, et al. [23]	2014	Oncofertility preservation techniques in (penile vibratory stimulation and electroejaculation).	Case report	Spinal Cord
24.	Kafetsoulis A, et al. [24]	2006	Oncofertility preservation techniques in (penile vibratory stimulation and electroejaculation).	Original article	Fertility and Sterility
25.	Fayomi AP, et al. [25]	2019	Oncofertility techniques for prepubertal males.	Original article	Science
26.	Shams A, et al. [26]	2017	Oncofertility techniques for prepubertal males.	Review article	Current Stem Cell Research and Therapy
27.	Lawson, et al. [27]	2015	Ethical concerns regarding implementation of oncofertility procedures.	Original article	Journal of Psychosocial Oncology
28.	Quinn GP, et al. [28]	2009	Ethical concerns regarding implementation of oncofertility procedures.	Original article	Journal of adolescent health
29.	Gardino SL, et al. [29]	2010	Ethical concerns regarding implementation of oncofertility procedures in children.	Chapter of a book	Cancer treatment and research book - Springer link
30.	Dolin G, et al. [30]	2010	Ethical concerns regarding implementation of oncofertility procedures.	Chapter of a book	Cancer treatment and research book
31.	Knapp CA, et al. [31]	2010	Barriers faced in delivery of oncofertility care.	Chapter of a book	Cancer treatment and research book
32.	Shimizu C, et al. [32]	2015	Improper delivery of care due to miscommunication between healthcare departments.	Original article	International journal of clinical oncology
33.	Gorman JR, et al. [33]	2012	Patient concerns with delivery of oncofertility care.	Review article	Journal of cancer survivorship
34.	Garvelink MM, et al. [34]	2013	Patient concerns with delivery of oncofertility care.	Review article	Journal of psychosomatic obstetrics and gynaecology
35.	Malhotra N, et al. [35]	2022	Referral practices followed by healthcare workers in North India	Case study	JBRA assist reprod
36.	Shimizu C, et al. [36]	2013	Measures to combat hurdles faced in oncofertility care.	Original article	Breast Cancer
37.	Jill Scott - Trainer, et al. [37]	2010	Measures to correct improper counseling.	Chapter of a book	Cancer treatment and research book
38.	Seline Tam, et al. [38]	2018	Educational aids via social media platforms and measures for financial concerns.	Review article	Journal of cancer education
39.	Hanselin MR, et al. [39]	2018	Educational aids and considerations regarding sociocultural issues when dealing with oncofertility care.	Review article	An American society of clinical oncology journal

## Conclusions

Oncofertility is a serious complication of cancer therapy, which causes patients distress and insecurity regarding their future, especially in the case of adolescent and women population groups. Oncofertility is an emerging discipline that is a necessity for the fertility preservation of such patients. This discipline mandates the coordination among various specialty groups in the domains of diagnosis, therapeutics, fertility preservation, endocrine, and reproductive health. In this article we have addressed the effects of cancer treatment imposes on fertility, the options available to cancer patients to preserve their fertility the various hurdles and challenges we face in delivering oncofertility care across different areas including challenges faced by the providers due to knowledge constraints and improper protocol for referral pathways, difficulties faced by patients due to improper counseling and lack of awareness. Ethical and social conflicts the patient and provider face while making such decisions and measures we can take to overcome these obstacles to improve the quality of life of the patient post-survival.
